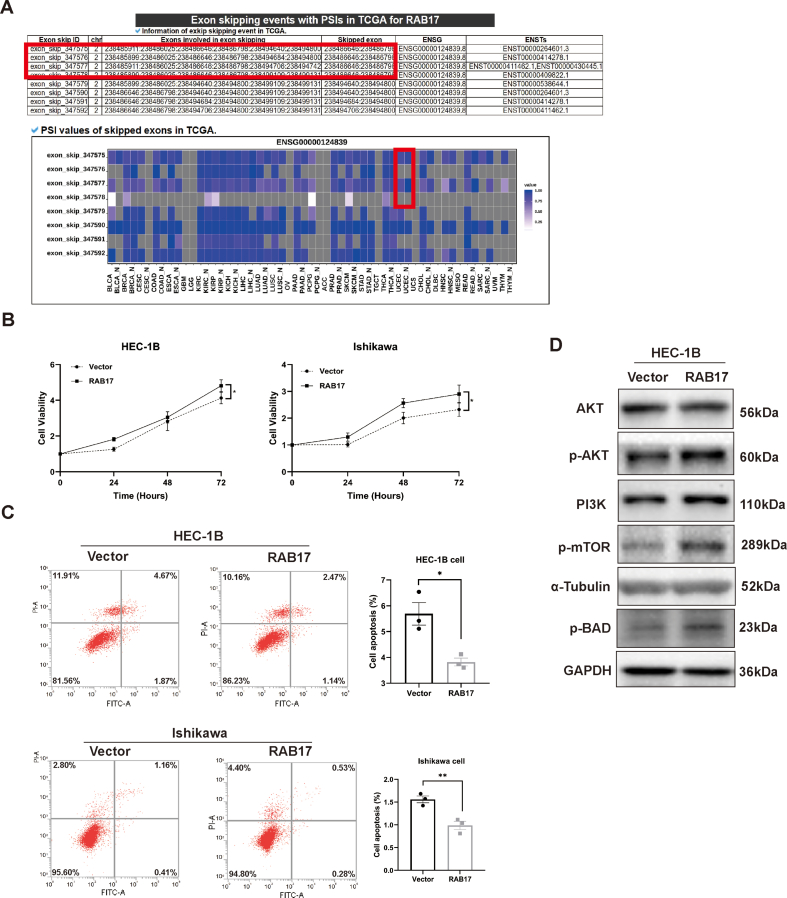# SNORD3B-2 promotes endometrial carcinoma progression by 2′-O-methylation modification of PLK1 and regulating RAB17 alternative splice

**DOI:** 10.1016/j.gendis.2024.101329

**Published:** 2024-05-17

**Authors:** Xi Chen, Yuping Du, Bumin Xie, Qianhui Li, Yumeng Ji, Yang Han, Xiujie Sheng, Shuo Chen, Yang Zhao

**Affiliations:** a Department of Obstetrics and Gynecology, The Third Affiliated Hospital of Guangzhou Medical University, Guangzhou, Guangdong 510150, China; b Department of Gynecologic Oncology Research Office, The Third Affiliated Hospital of Guangzhou Medical University, Guangzhou, Guangdong 510150, China; c Guangzhou Key Laboratory of Targeted Therapy for Gynecologic Oncology, The Third Affiliated Hospital of Guangzhou Medical University, Guangzhou, Guangdong 510150, China; d Guangdong Provincial Key Laboratory of Major Obstetric Diseases, The Third Affiliated Hospital of Guangzhou Medical University, Guangzhou, Guangdong 510150, China; e Guangdong Provincial Clinical Research Center for Obstetrics and Gynecology, The Third Affiliated Hospital of Guangzhou Medical University, Guangzhou, Guangdong 510150, China; f Guangdong-Hong Kong-Macao Greater Bay Area Higher Education Joint Laboratory of Maternal-Fetal Medicine, The Third Affiliated Hospital of Guangzhou Medical University, Guangzhou, Guangdong 510150, China

Endometrial cancer (EC) is one of the most common gynecological malignant tumors. Further investigation of the potential molecular mechanism of EC is important. Small nucleolar RNA (snoRNA) is a type of non-coding RNA, with an unclear biological function in EC. We found that Box C/D snoRNA SNORD3B-2 participated in EC oncogenesis and development via the PI3K-AKT signaling pathway. RNA immunoprecipitation (RIP) revealed that SNORD3B-2 bound to polo-like kinase 1 (PLK1) through fibrillin (FBL). RTL-P (reverse transcription at low dNTPs-PCR) and actinomycin D assays confirmed that SNORD3B-2 directed 2′-O-methylation modification of PLK1 mRNA, and the modification could promote the stability of PLK1 mRNA which could mediate tumor growth and metastasis in EC. Moreover, SNORD3B-2 overexpression was associated with retained Exon 3 of RAB17 thus activating the PI3K-AKT signaling pathway. This alternative splicing was achieved by SNORD3B-2 regulating the protein level of splice factor SF3B1 (splicing factor 3b subunit 1).

In this study, we searched for the TCGA database and EC tissues from patients at our hospital. We found abnormal expression of SNORD3B-2 in the endometrial carcinoma tissue (Fig. 1A and Table S1). Kaplan–Meier analysis indicated that EC patients with a high level of SNORD3B-2 expression had significantly poor recurrence-free survival (Fig. 1B). These data suggested that SNORD3B-2 played an important role in EC tumorigenesis and progression. To determine the role of SNORD3B-2 in EC, we constructed the SNORD3B-2 plasmid and ASO-SNORD3B-2 for cell function experiments and found that SNORD3B-2 promoted cell proliferation and invasion and inhibited apoptosis in both HEC-1B and Ishikawa cells (Fig. 1C; Figs. S1A–D). In *in*-*vivo* experiments with nude mice, SNORD3B-2 was observed to enhance tumor growth *in vivo* (Fig. 1D; Fig. S1E). Further, patient-derived organoids preserved the pathologic and molecular properties of cancer. To initially explore the therapeutic potential of SNORD3B-2, we built a human EC organoid model (ECO). We found the growth of ECOs was promoted in the LV-SNORD3B-2 group (Fig. 1D; Fig. S2). To improve the binding affinity and nuclease resistance, ASOs targeting SNORD3B-2 were modified with 2′-O-methylation and cholesterol. ECOs were treated with modified ASOs and the growth of ECOs was inhibited (Fig. 1E; Fig. S2).

**Figure 1:**
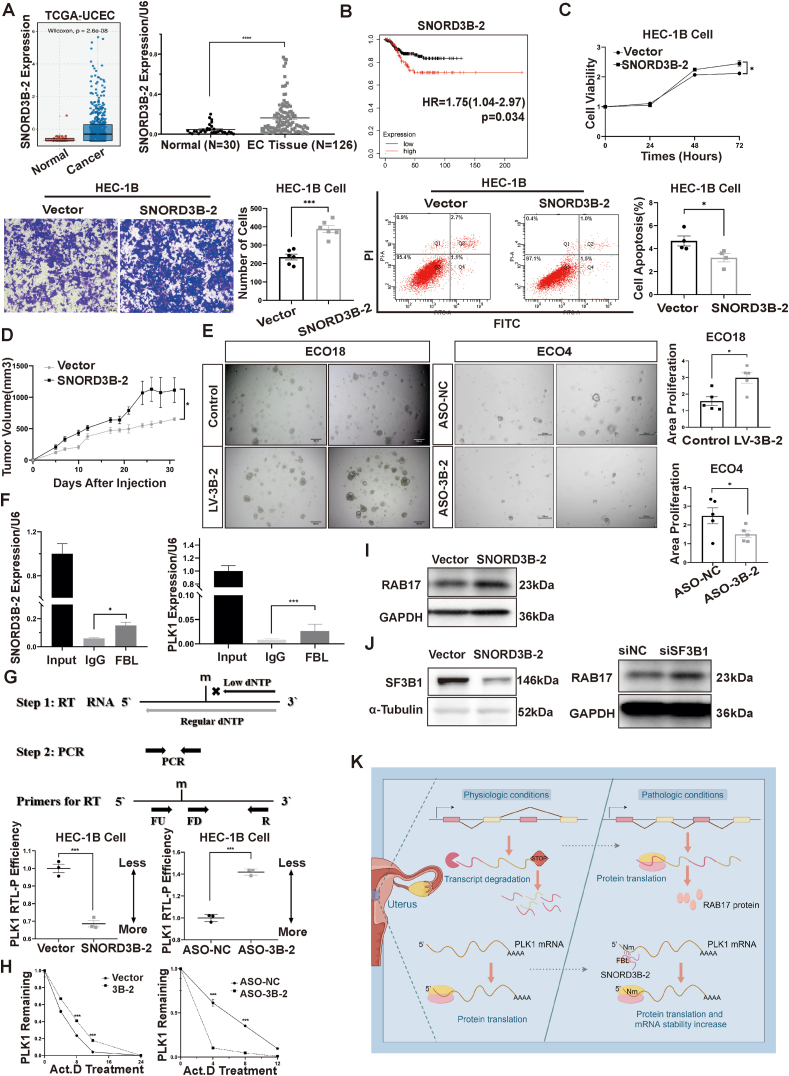
SNORD3B-2 regulates EC progression by directing the 2′-O-methylation modification of PLK1 and influences the alternative splicing of RAB17 through SF3B1. **(A)** Expression of SNORD3B-2 in TCGA-UCEC database and tissues from patients with EC. **(B)** Kaplan−Meier RFS analysis for EC patients with low and high SNORD3B-2 expression. **(C)** Cell proliferation, invasion, and apoptosis were detected by CCK8, transwell, and flow cytometry assays. **(D)** Tumor growth curve in nude mice. **(E)** Represent images of EC organoids derived from patients with SNORD3B-2 transfection or ASO targeting SNORD3B-2. **(F)** RNA immunoprecipitation (RIP) analysis showed SNORD3B-2 bound to FBL directly. RIP assay showed PLK1 mRNA enriched on FBL protein. **(G)** Graphical representation of RTL-P method. RQ means relative quantity. The level of 2′-O-methylation modification on PLK1 mRNA caused by SNORD3B-2 was detected by RTL-P assay. **(H)** Actinomycin D treatment was employed for the effect of SNORD3B-2 and 2′-O-methylation modification on PLK1 mRNA stability. **(I)** Western blot showed the change of RAB17 caused by SNORD3B-2. **(J)** SF3B1 expression was inhibited by SNORD3B-2. The expression of RAB17 increased after SF3B1 knockdown. **(K)** Schematic diagram of SNORD3B-2 that regulated PI3K-AKT signaling pathway by directing the 2′-O-methylation modification of PLK1 and influencing the alternative splicing of RAB17 (by Figdraw). EC, endometrial cancer; RFS, recurrence-free survival; PLK1, polo-like kinase 1; SF3B1, splicing factor 3b subunit 1.

Next, to explore the mechanism of SNORD3B-2 in EC, transcriptome-wide RNA sequencing was performed. SNORD3B-2 activated multiple tumor-related signaling pathways, including the PI3K-AKT signaling pathway (Fig. S3A). Western-blot assay confirmed PI3K-AKT signaling pathway was activated by SNORD3B-2 (Fig. S3B). This activation was eliminated following ASO targeted SNORD3B-2 (Fig. S3B). Previous studies indicated that SNORDs could guide 2′-O-ribose methylation modifications which were catalyzed by the enzyme FBL via antisense complementarity.^1^ In this study, RIP-PCR showed that SNORD3B-2 combined with FBL (Fig. 1F). Subsequently, we employed 2′-O-methylation sequencing and BLAST (basic local alignment search tool) to search for the potential methylation modification targets and found PLK1 was the candidate target. It is reported that PLK1 is highly expressed in EC, which is closely related to a poor prognosis, pathological stage, and clinical grade.^2^ RIP-PCR was conducted and PLK1 mRNA was proved to be enriched on methyltransferase FBL protein (Fig. 1F). As 2′-O-methylation sites could reduce RTL-P efficiency,^3^ we designed specific primers and found that SNORD3B-2 led to higher 2′-O-methylation modification level of PLK1 mRNA and in the contrast, knockdown of SNORD3B-2 led to lower 2′-O-methylation modification level of PLK1 mRNA (Fig. 1G). It was reported that 2′-O-methylation modification was involved in RNA stability,^4^ thus we then assayed mRNA stability with actinomycin D to block transcription. A significant increase of PLK1 mRNA stability in SNORD3B-2 versus control was observed. Consistent with the results above, the stability of the PLK1 mRNA in the siFBL group was lower compared with the siNC group (Fig. 1H). We found that overexpression of SNORD3B-2 increased the expression level of PLK1 protein, while knockdown of SNORD3B-2 reduced the expression level of PLK1 protein (Fig. S3C). Consistently, SNORD3B-2 overexpression also significantly increased the level of PLK1 expression in subcutaneous transplantation tissues (Fig. S3D). We further evaluated whether PLK1 inhibition could rescue the effects of SNORD3B-2 overexpression on the biological behavior of EC cells. Knockdown of PLK1 or FBL significantly reversed the influence of SNORD3B-2 on EC cell proliferation and apoptosis (Fig. S3E–G).

We analyzed RNA sequencing data and found that SNORD3B-2 affected a series of alternative splicing events (skipped exons). As reported, the splice variant contained in Exon 3 of RAB17 (RAB17 E3^+^) can encode a protein, while another variant lacking Exon 3 (RAB17 E3^−^) is a candidate gene for nonsense-mediated mRNA decay, which is degraded after its production. To explore whether SNORD3B-2 affected the alternative splicing of RAB17, we also looked up alternative splicing form RAB17 E3^+^ in the TCGA database and found that it was elevated in EC tissues (Fig. S4A). The level of RAB17 was elevated by SNORD3B-2 in the EC cells (Fig. 1I). Overexpression of RAB17 promoted cell proliferation and inhibited apoptosis in EC (Fig. S4B–D). In addition, similar to those results reported by others,^5^ overexpression of RAB17 in EC cells activated the PI3K-AKT signaling pathway (Fig. S4E). We further examined common splice factors that SNORD3B-2 could affect. We found that SNORD3B-2 inhibited the expression of SF3B1 protein. When the SF3B1-targeted siRNA transfected into cells to reduce the SF3B1 protein level, the level of RAB17 protein was also inhibited (Fig. 1J).

EC is one of the major malignant tumors threatening women's health. Patients with advanced, relapsed, or metastatic EC have poor prognosis, so it is important to explore related molecular mechanisms and find targeted therapeutic targets. In this study, SNORD3B-2 was found to be associated with poor prognosis of EC. SNORD3B-2 was overexpressed in EC and led to malignant biological behavior of EC. This study revealed that SNORD3B-2 affected an important EC pathway, the PI3K-AKT pathway, by directing the 2′-O-methylation modification of PLK1 and influencing the alternative splicing of RAB17 through SF3B1. In addition, based on ECO models, this study identified the potential of SNORD3B-2 as an EC therapeutic target.

## Ethic declaration

All experiments involving animals were conducted according to the ethical policies and procedures approved by the Ethics Committee of Guangdong Medical Laboratory Animal Center (Approval No. B202008-10). All patients provided consent for the research use of the collected tissues, which was approved by the Ethics Committee of Guangzhou Medical University (No. 2020–066).

## Conflict of interests

The authors are grateful to Figdraw for offering an online drawing platform.

## Funding

This work was supported by the 10.13039/501100001809National Natural Science Foundation of China (No. 82072854 to Yang Zhao, 82272985 to Shuo Chen), the 10.13039/501100015806Project for Key Medicine Discipline Construction of Guangzhou Municipality, Guangdong, China (No. 2021-2023-17), and the Science and Technology Projects in Guangzhou, Guangdong, China (No. 202201020093 to Yang Zhao).

## Figures and Tables

**figs1:**
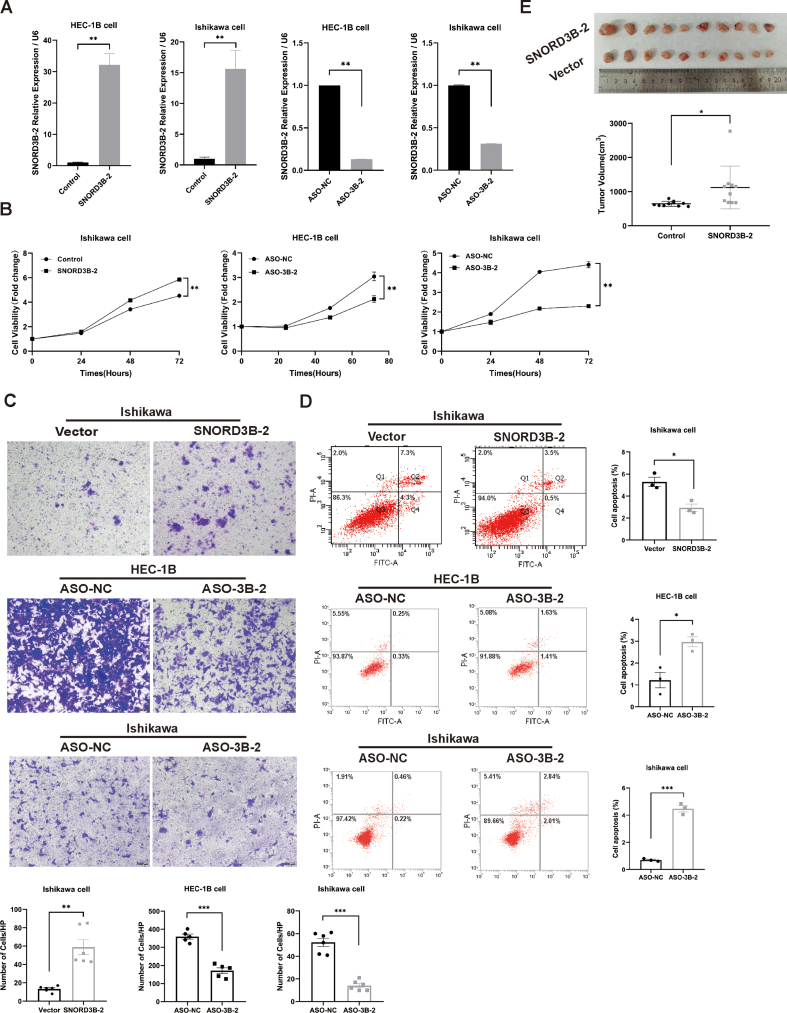


**figs2:**
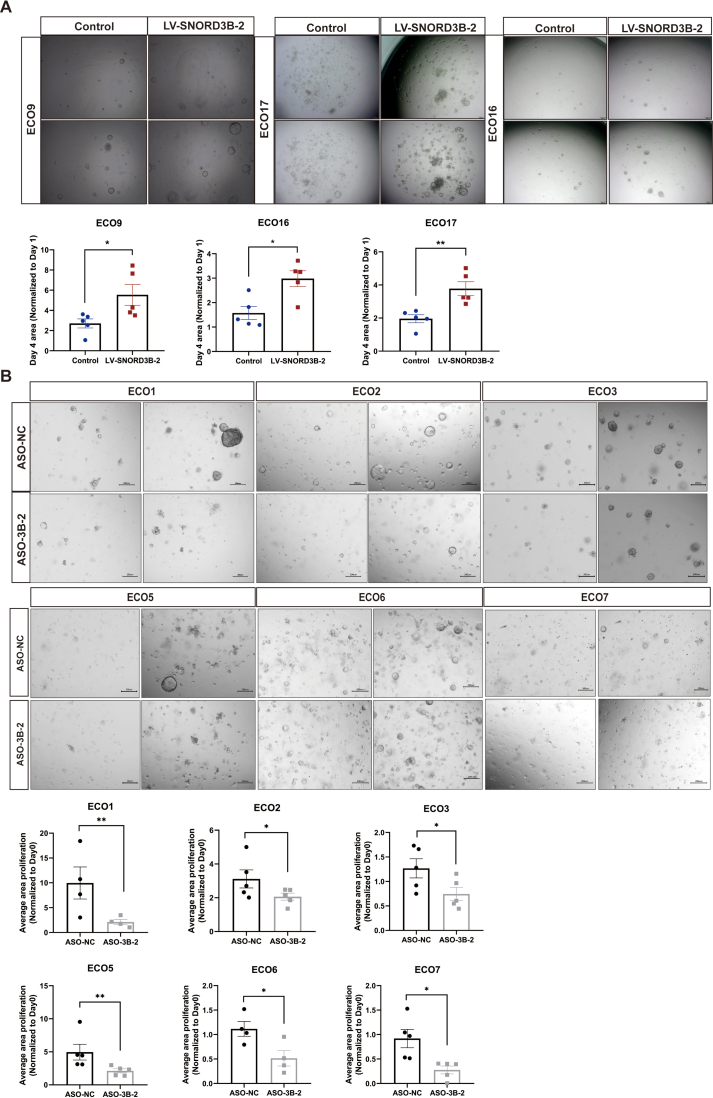


**figs3:**
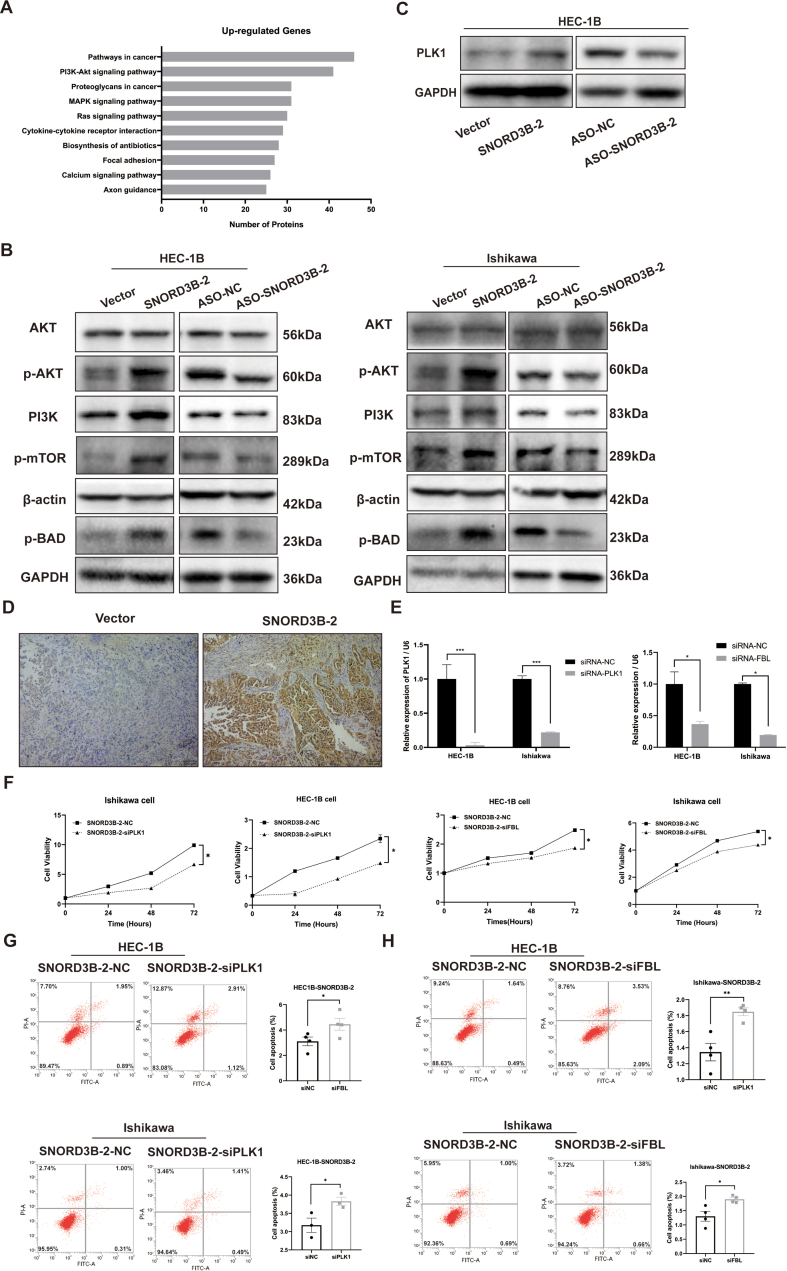


**figs4:**