# Supplementary material for: SNORD3B-2 promotes endometrial carcinoma progression by 2′-O-methylation modification of PLK1 and regulating RAB17 alternative splice
Source: Genes Dis. 2024 May 17;12(2):101329. doi: 10.1016/j.gendis.2024.101329 (PMC11616056; doi:10.1016/j.gendis.2024.101329)
Supplement: Multimedia component 1 [file mmc1.docx]

**Materials and Methods**

**Clinical tissues**

Clinical tissue samples were obtained from patients undergoing surgery for endometrial cancer in the Department of Gynecology, the Third Affiliated Hospital of Guangzhou Medical University. The normal control was obtained from patients who underwent total hysterectomy for hysteromyoma. All the cases have never been treated with radiotherapy, chemotherapy, surgery and cellular immunotherapy before the operation, and no other tumors were combined. The experiments were approved by Ethics Committee of the Third Affiliated Hospital of Guangzhou Medical University, and the informed consent form was signed by the patient.

**Cell lines and culture**

HEC-1A, HEC-1B, Ishikawa, hESC, hEEC cells were purchased from ATCC. Cells were cultured in RPMI 1640 or Dulbecco's Modified Eagle Media (DMEM) with 10% foetal bovine serum (FBS; Gibco, NY, USA) and 1% antibiotic/antimycotic solution (Solarbio, Beijing, China) at an atmosphere of 37°C with 5% CO2 according to manufacturer`s instructions.

**Construction of plasmids, ASOs and siRNAs and transfection**

The SNORD3B-2 overexpression vector was constructed by syngentech (Beijing, China). All of the siRNAs and ASOs were purchased from Ribobio (Guangzhou, China). Cells were transfected with siRNA or ASOs at 50%-60% confluence using Lipofectamine 3000 (Invitrogen) according to the manufacturer’s protocol. HEC-1B cells and Ishikawa cells were grown to 90% confluence before being transfected with plasmids using Lipofectamine 3000 (Invitrogen) according to the manufacturer’s protocol. Negative control siRNA is provided by Ribobio (Guangzhou, China).

Sequences of siRNAs: siRNA-FBL (5'-3') GGGCTAAGGTTCTCTACCT, siRNA-SF3B1 (5'-3') GATGACTATTCATCATCTA, siRNA-PLK1 (5'-3') CCTTAAATATTTCCGCAAT. ASO-SNORD3B-2: CCGAGGAAGAGAGGTAGCGT.

**Xenografts experiments**

4–6-week-old BALB/c female nude mice were purchased from Guangdong Medical Laboratory Animal Center (Guangdong, China). The animals were randomly divided into two groups and a total of 5 × 10^6^ HEC-1B cells overexpressing SNORD3B-2 or vector were injected subcutaneously. The long and short diameters of the subcutaneous tumors were measured every 3 days. Tumor volume was calculated as: V (volume) = (length × width^2^)/2. Mice were sacrificed at day 33. Tumors were harvested and collected in a proper way for subsequent analysis. All animal experiments were approved by the Animal Experimental Ethics Committee of Guangdong Medical Laboratory Animal Center.

**EC** **organoid model**

Human EC organoids were constructed as described previously to simulate the microenvironment of the human body[14]. Considering the differences in individual responses to drugs, organoids from the same patient were randomly divided into experimental group and control group. Lentivirus of SNORD3B-2 was transfected into the organoids to facilitate our investigation of the role played by SNORD3B-2 in endometrial cancer progression. ASO targeting SNORD3B-2 or ASO-NC were added into the EC organoids to detect the therapeutic effect of targeting SNORD3B-2. Human EC organoid growth was observed daily by microscope. Organoids with initial diameter interval of 100±50μm were selected for statistical analysis according to avoid selection bias caused by different initial volume. We used organoid area to measure the impact of experimental treatment on organoid proliferation, and used blind data collection with different researchers in charge of organoid grouping and measurement was adopted to limit information bias. The experimental group and the control group were changed and dosing at the same time. We selected a total of 9 cases organoid for study, which was the maximum sample size that can meet the inclusion criteria during the study. The sample size was relatively limited.

Patient-derived organoids can better mimic the key characteristics of parental tumors in terms of molecular properties and tumor heterogeneity, which is a suitable model for drug preclinical experiments. Nevertheless, the results of preclinical studies need to go through procedures including pharmacology, pharmacokinetics, toxicology, long-term toxicity tests, and phase trials of clinical studies before they can be finally applied to patients. Although this study confirmed the therapeutic potential of SNORD3B-2 in ECOs, there was still a long way to go before clinical application.

**Cell viability and colony formation assays**

Cell proliferation was analyzed using CCK-8 experiment. Cells were seeded into 96-well plates at a density of 5×10^3^/ well and cultured at 37°C and 5%CO_2_. 10ul of CCK-8 reagent (Yeasen, Shanghai, China) was added to every well in proper times and incubated at 37°C for 2h. The absorbance was measure according to that instruction at a wavelength of 450 nm.

500 cells of different groups were seeded in 6-well plate and cultured with medium containing 10% FBS for approximately 12 days. Mediums were changed every 5 days. After 12 days, colonies were stained with 0.1% crystal violet (Vicmed, China) after being fixed with methanol. The colony formation rate was calculated by the number of stained colonies.

**Organoid viability assay**

Cell Counting-Lite 3D Luminescent Cell Viability Assay (Vazyme, # DD1102) was used to measure organoid cell viability. Organoid growth assays were performed in 96-well plates. Organoids were transfected with lentivirus of SNORD3B-2 or negative control before counted manually and seeded out at uniform density of 10-20 organoids per well. The media was replenished every second day. Organoids seeded in the 96-well plate were exposed to 100 μl Cell Counting-Lite 3D in culture medium for 30 min at room temperature according to the manufacturer’s instruction at the end point of the experiment.

**Flow cytometric analysis**

Cell apoptosis was measured by a flow cytometer (FACSCalibur, BD, USA). Based on the manufacturer’s guidelines, cells of different groups were collected and then stained with FITC and PI (BD Biosciences, San Jose, CA, USA). Then flow cytometry was used to analyze the apoptosis rate. The cell apoptosis data were analyzed by flow cytometry (FACS Calibur, BD Biosciences, San Jose, CA, USA). Each experiment was performed three times.

**Cell invasion assay**

Transwell assay was used for measurement of cell invasion. Firstly, 30 μL of Matrigel (BD Bioscience, San Jose, CA, USA) were precoated in the chambers (Corning, USA) for 4h. 5×10^4^ cells were suspended in medium without FBS and seeded into the upper compartment of a 24-well chamber. Medium containing 10% FBS was added to the lower chambers as a chemoattractant. The cells were incubated for another 48 h. cells on the upper surface of the membrane were removed with cotton swabs. The cells on the lower side of the membrane were fixed with 4% methanol, stained with 0.05% crystal violet. The number of invasion cells was calculated from three independent experiments.

**RNA extraction, quantitative real-time PCR (qRT-PCR) and PCR**

Total RNA was extracted from the cells using Trizol reagent (Takara, Japan) and the purity and concentration of RNA were determined using Thermo Scientific NanoDrop2000 (Thermo Fisher Scientific, USA). Then, 5μg total RNA was reversely transcribed into cDNA using PrimeScript-RT kit (Takara Bio, Japan). The qRT-PCR reaction was performed using the SYBR Select Master Mix kit (Yeasen, China). U6 was used as the internal reference, and the relative expression amount was calculated by 2 −^△△^ Ct method.

For the detection of RAB17 mRNA variants, a mixture of oligo-dT (Sigma-Merck, Darmstadt, Germany) and primers were used for amplification. PCR products were separated using electrophoresis on a 2% agarose gel. Band intensity was calculated using ImageJ (NIH, Bethesda, MD, USA).

The primers used in the study are as follows: SNORD3B-2 (forward: 5'-TTCAGCGGTGACGGCTCT-3', reverse: 5'-GGGAAACGGCGACAAAA-3'), RAB17 (forward: 5′‐GTGGGCAACAAGACGGACCTCAG‐3', reverse: 5'- CTCGCGGGCCCCTTGTTCAG-3'), PLK1 (forward: 5′‐ATTACATAGCTCCCGAGGT‐3', reverse: 5'- CAAGGTATACATGATACACCCA -3'), U6 (forward: 5′‐CTCGCTTCGGCAGCACA‐3', reverse: 5'- AACGCTTCACGAATTTGCGT -3').

**RTL-P assay**

The procedure was generated from the RTL-P method described previously[15]. 5μg total RNA and specific oligo-dT RT primer were incubated together under two different conditions: (a) standard reverse transcription of 1mM dNTP; (b) low dNTP concentration (0.1mM). cDNA was generated and RT-PCR was performed in 20μl reactions. Relative quantity of PLK1 mRNA (PLK1 RTL-P efficiency, RQ) was first normalized with internal reference gene (U6) and then with the high dNTP. The value could be finally compared between different experimental groups.

**Western blot and Antibodies**

The cells of each treatment group were collected for lysis and total protein was extracted. Proteins of different molecular weights were separated by SDS-PAGE with 10% polyacrylamide gel electrophoresis and subsequently transferred to a PVDF membrane (Millipore). Blocking was performed with 3% BSA for 2h and the primary antibody was incubated overnight at 4°C. The PVDF membrane was washed with TBST for 6×5 min, followed by incubation with secondary antibody (1: 8000) for 1-2 h at room temperature, and then wash with TBST for 3×5 min. Subsequently, the color was developed in the chemiluminescence detection system using ECL luminescence detection kit.

The experiment was performed with commercial antibodies, PLK1 (Proteintech, 10305-1-AP), RAB17 (Proteintech, 17501-1-AP), AKT (Proteintech, 10176-2-AP), p-AKT (Phospho-AKT (Ser473), Proteintech, 66444-1-Ig), PI3K (Cell signaling technology, 4249S), p-mTOR (Proteintech, 67778-1-Ig), p-BAD (Cell signaling technology, 9291S), β-actin (Proteintech, 20536-1-AP).

**Immunohistochemistry (IHC)**

Standard procedures were performed. Briefly, paraffin-embedded tissues were cut into 4 um sections and de-chained and rehydrated in a bath of xylene and alcohol. Antigen repair was performed in 0.01 M citrate buffer (pH 6.0) with a microwave at 98°C for 5 min. Staining was performed using immunohistochemical staining kit (Bioss, SP-0022, Beijing, China) after the slides were then cooled to room temperature. All of the protocols were in accordance with the manufacturer's recommendation. For the PLK1 antibody, a dilution of 1:100 was used. After DAB (Abcam, USA) coloration, counterstaining was performed with hematoxylin (Solarbio, Beijing, China). The IHC staining in these specimens was scored by two observers under the Olympus CX31 microscope (Olympus, Center Valley, PA).

**RNA-binding protein immunoprecipitation (RIP) assay**

Cells were lysed in RIP lysis buffer with protease and RNase inhibitors. The cell lysates were incubated with beads (Bimake, USA) and primary antibodies or IgG at 4 °C overnight. Then, the beads were washed with TBST and incubated with proteinase K to remove proteins. The immunoprecipitated RNA extracts were reverse-transcribed and analyzed by qRT-PCR.

**RNA-sequence**

Cells transfected with SNORD3B-2 and vector plasmid were collected and total RNA was extracted using Trizol (Takara, Japan). The sequencing libraries were constructed using Neb Next Ultra RNA Library Prep Kit for Illumina (Neb, USA) according to the manufacturer's recommendations. Briefly, mRNA is purified, cleaved at elevated temperature, and cDNA is synthesized. The library fragments were purified using AMPure XP system (Beckman Coulter, Beverly, USA) and cDNA fragments with a length of 150–200 bases were screened. The polymerase chain reaction was carried out using polymerase, universal primer and index primers. Finally, PCR products were purified (AMPure XP system) and the library was evaluated for quality on the Agilent Bioanalyzer 2100 system.

**2`-O-Methylation sequence**

NM-Seq service was provided by CloudSeq Biotech Inc. (Shanghai, China). Briefly, RNA Fragmentation Reagents (Thermo Fischer Scientific) was added in the RNA samples and were fragmented at 95 °C for 5 min. 3′-end of RNA fragments were repaired with Antarctic phosphatase (New England BioLabs) at 37 °C for 30 min. Then, repaired RNA samples were oxidized/eliminated using 10 mM NaIO_4_ (Sigma-Aldrich) with 200 mM lysine-HCl buffer (pH 8.5, Sigma-Aldrich) in a total volume of 40 µl at 37 °C for 30 min. After eight cycles of oxidation–elimination–dephosphorylation being performed, A final round of oxidation/elimination reaction was performed, excluding dephosphorylation. Then, purified RNA samples were 5′ phosphorylated by T4 polynucleotide kinase 3′ phosphatase minus (New England BioLabs) at 37 °C for 60 min. Libraries were constructed using NEBNext Small RNA Library Prep Set for Illumina (New England BioLabs). Sequencing was carried out on Illumina HiSeq4000 according to the manufacturer’s instructions.

**Actinomycin D assay**

Cells were seeded in 6-well plate overnight and then transfected. 24 h later, cells were then exposed to 2 mg/L actinomycin D (Selleck) for 0, 2, 4, 8, 12 and 24 hours. The cells were harvested and the stability of PLK1 mRNA was analyzed using quantitative reverse transcription PCR (qRT-PCR).

**Statistics**

GraphPad Prism v8.0 software (GraphPad Software, La Jolla, CA, USA) was used for comparisons between different groups. Comparisons between two groups were conducted by the two-tailed Student’s t-test. The results are presented as the mean ± standard deviation. P < 0.05 was considered statistically significant; *P < 0.05, **P < 0.01, ***P < 0.001.

**Table S1 Correlation of SNORD3B-2 expression with different clinicopathological features of endometrial carcinoma.**

Italics means P < 0.05.

**Supplementary Figures**

**
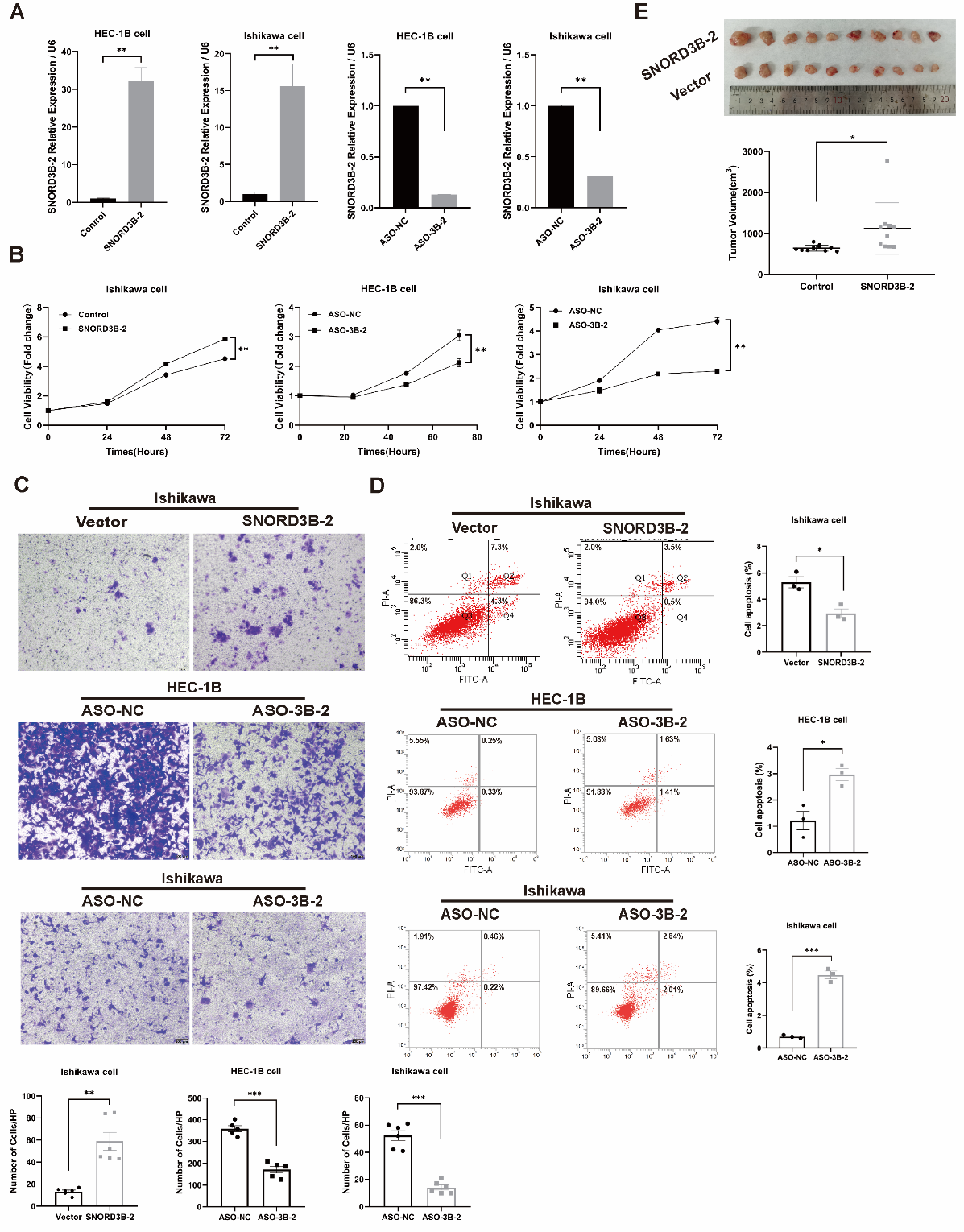
**

**Figure S1** SNORD3B-2 promotes the proliferation and invasion of EC both in vitro and in vivo. **(A)** Level of SNORD3B-2 in Ishikawa and HEC-1B cells after transfection of SNORD3B-2 plasmid and ASO-SNORD3B-2, as determined by qRT-PCR (normalized to U6). (**B)** Cell proliferation capability detected by CCK8 assay. (**C)** Detection of the invasion capability of HEC-1B cells transfected with SNORD3B-2 plasmid or ASO-SNORD3B-2 by transwell assay. (**D)** Detection of the apoptosis of HEC-1B cells transfected with SNORD3B-2 plasmid or ASO-SNORD3B-2 by flow cytometry assay. (**E)** Images of tumor formation in nude mice injected subcutaneously with HEC-1B cells over-expressing SNORD3B-2 or vector (upper side) after 31 days. Tumor-bearing mice of different group were sacrificed and tumors excised from them were shown. Growth curve of tumor volume and tumor volume from different group were shown.


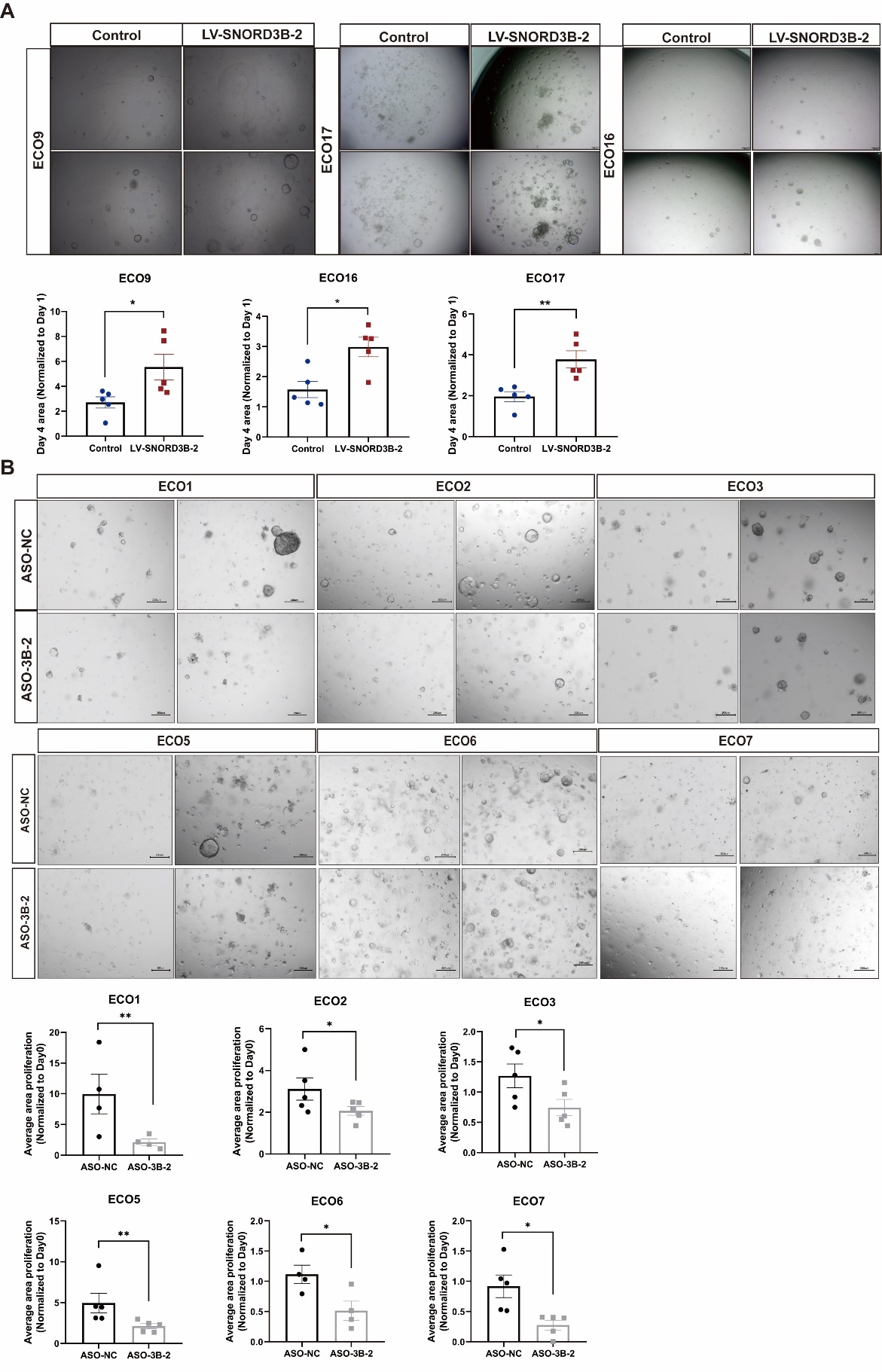


**Figure S2** SNORD3B-2 promotes EC organoid model proliferation. **(A)** Images of EC organoids derived from 3 patients with or without SNORD3B-2 lentivirus transfection. Representative images under 4x magnification are shown at the left. Scale bar, 200 μm. (**B)** Images of 6 ECOs cases with or without ASO-SNORD3B-2 treatment. Area of signal organoid model was calculated and normalized with the area of Day0.


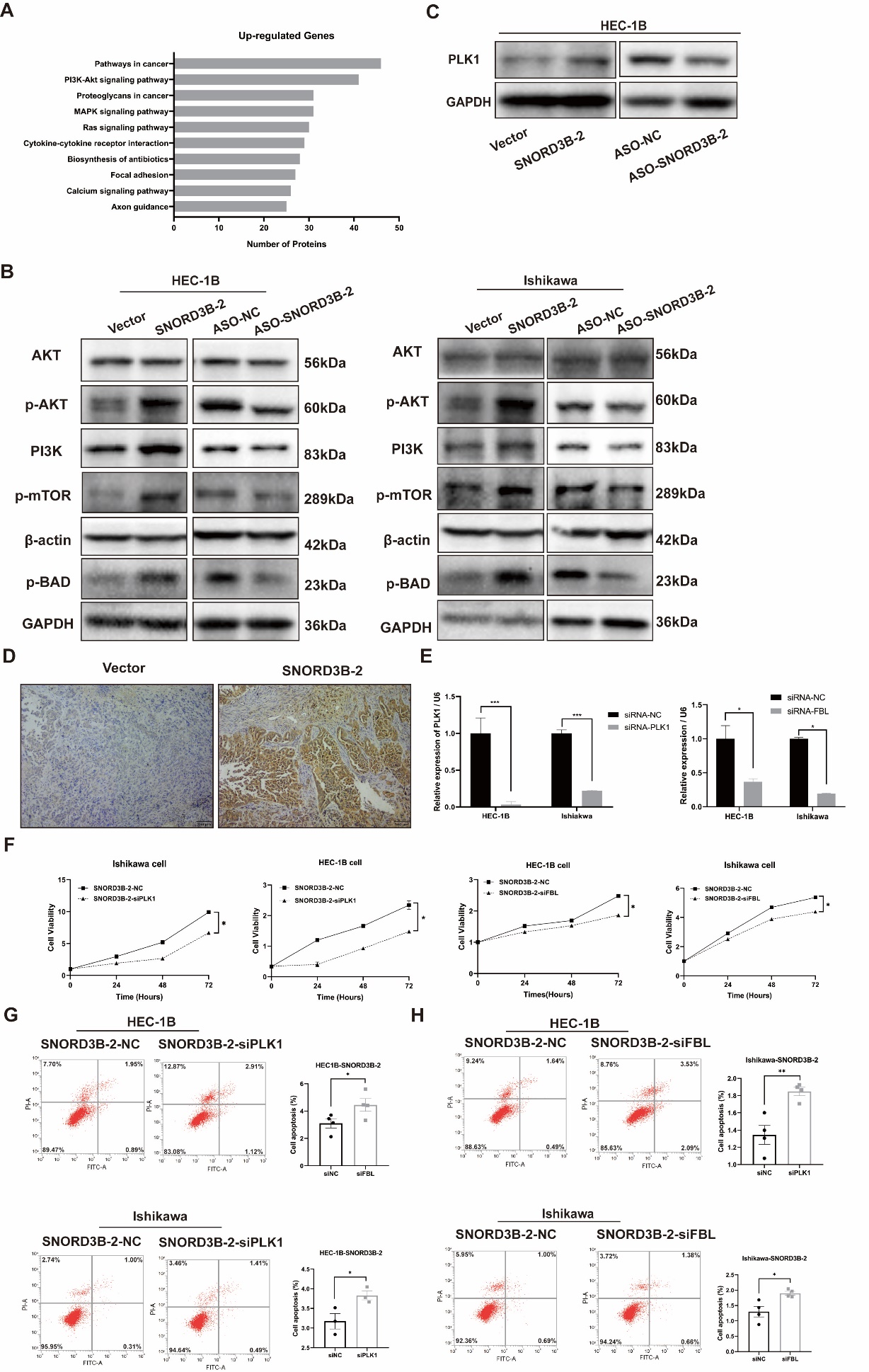


**Figure S3** SNORD3B-2 regulates PI3K/AKT signaling pathway via directing 2`-O-methylation modification of PLK1. **(A)** KEGG pathway analysis for the up-regulation mRNAs of RNA-seq showed PI3K/AKT pathway activation. (**B)** Abnormal activation of p-AKT, PI3K, p-mTOR, and BAD signaling was identified in HEC-1B cells overexpressing SNORD3B-2. The activation was abolished following treatment with ASO targeting SNORD3B-2. (**C)** Western blotting analysis of PLK1 expression derived from endometrial cancer cells with SNORD3B-2 overexpression or knockdown. (**D)** IHC showed PLK1 up-regulated in xenografts of nude mice. Scale bars: 100 μm. (**E)** Establishment of PLK1-knockdown cells and FBL-knockdown cells. (**F, G)** Knockdown of FBL could partially retain the tumorigenesis function of SNORD3B-2 on proliferation and apoptosis.


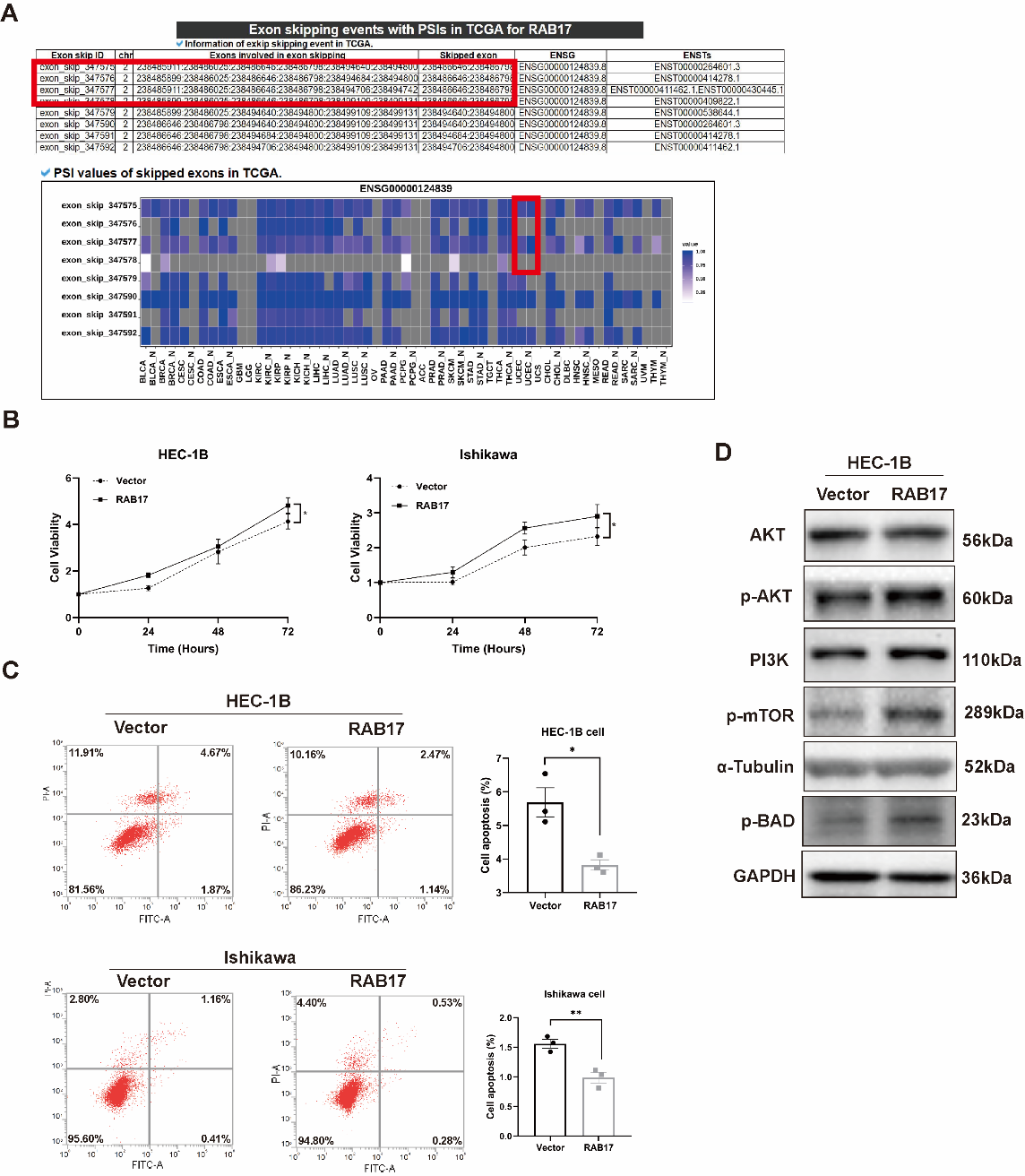


**Figure S4** SNORD3B-2 regulates PI3K/AKT signaling pathway via affecting RAB17 alternative splicing through SF3B1. **(A)** RAB17 alternative splicing form in the TCGA database showed RAB17 E3+ content is higher in endometrial cancer tissues. (**B, C)** Cell proliferation increased while apoptosis decreased due to overexpressing of RAB17. (**D)** Western-blot showed RAB17 E3+ activates PI3K/AKT pathway.
